# Epigenomics and transcriptomics profiles of developing zebrafish heart cells

**DOI:** 10.1038/s41597-025-05895-9

**Published:** 2025-10-07

**Authors:** Gulrez Chahal, Michael P. Eichenlaub, Markus Tondl, Michał Pawlak, Monika Mohenska, Lin Grimm, Lauren Bottrell, Mark Drvodelic, Sara Alaei, Jeannette Hallab, Lisa N. Waylen, Jose M. Polo, Cédric Blanpain, Nathan Palpant, Fernando J. Rossello, Minna-Liisa Änkö, Peter D. Currie, Benjamin M. Hogan, Cecilia Winata, Ekaterina Salimova, Hieu T. Nim, Mirana Ramialison

**Affiliations:** 1 Australian Regenerative Medicine Institute and Systems Biology Institute Australia, Monash University, Clayton, Victoria 3800 Australia; 2 Murdoch Children’s Research Institute, Royal Children’s Hospital, Parkville, Victoria 3052 Australia; 3 The Novo Nordisk Foundation Center for Stem Cell Medicine (reNEW) Melbourne, Melbourne, Australia; 4 Institute of Hematology and Blood Transfusion, Warsaw, Poland; 5 Adelaide Centre for Epigenetics, Faculty of Health and Medical Sciences, The University of Adelaide, Adelaide, SA 5000 Australia; 6 South Australian immunoGENomics Cancer Institute, Faculty of Health and Medical Sciences, The University of Adelaide, 5000 Adelaide, SA Australia; 7 Institute for Molecular Bioscience, The University of Queensland, Brisbane, Queensland 4072 Australia; 8 Division of Genomics of Development and Disease, Institute for Molecular Bioscience, The University of Queensland, St Lucia, 4072 QLD Australia; 9 Interdisciplinary Research Institute (IRIBHM), Université Libre de Bruxelles (ULB), Bruxelles, 1070 Belgium; 10 Faculty of Medicine and Health Technology, Tampere University, Tampere, 33100 Finland; 11 Hudson Institute of Medical Research, Melbourne, Victoria 3168 Australia; 12 Peter MacCallum Cancer Centre, Melbourne, VIC 3000 Australia; 13 Department of Anatomy and Physiology and the Sir Peter MacCallum Department of Oncology, University of Melbourne, Melbourne, VIC 3000 Australia; 14 International Institute of Molecular and Cell Biology in Warsaw, Warsaw, Poland; 15 Department of Paediatrics, Royal Children’s Hospital, MDHS Faculty, Flemington Road, The University of Melbourne, Melbourne, Victoria 3010 Australia

**Keywords:** Disease model, Gene regulatory networks

## Abstract

*cis*-Regulatory elements (cREs) are essential for the spatio-temporal control of gene expression during development and disease. However, cRE activity is highly dependent on cell and tissue type. The developing heart is composed of several cell-types, predominantly cardiomyocytes. Therefore, cardiomyocyte-specific modelling is required to understand the *cis*-regulation of the developing heart. Zebrafish are an ideal model to study heart development, as they share several physiological features with the human heart during cardiogenesis. Here, we present a comprehensive cardiomyocyte-specific repertoire of cREs isolated from zebrafish larvae. This data combines *in vivo* transcriptomics and epigenetic profiling, providing insights into cREs and their associated genes involved in heart development. We further perform transgenic reporter assays for the identified cREs associated with *popdc2* and *bmp10* genes, validating these genomic regions as cardiac regulatory elements. We share this comprehensive, reproducible cardiomyocyte-specific cREs resource as an interrogable web tool for understanding the epigenetic and transcriptomic mechanisms underlying heart development and emergence of congenital heart defects.

## Background & Summary

*cis*-Regulatory elements (cREs), such as enhancers, promoters, insulators, and silencers, are essential for the spatio-temporal control of gene expression during development^1,2^. Multiple studies have shown that mutations in regulatory regions disrupt embryogenesis^3,4^ and play an important role in disease pathogenesis^5^. In contrast to protein-coding regions, *c*REs are more challenging to identify in the genome. Nonetheless, *c*REs can be indirectly identified as genomic regions associated with specific histone and chromatin modifications.

Several *c*REs have been identified as having a role in heart development and disease. These cardiac-relevant *c*REs were identified with chromatin immunoprecipitation, using antibodies against specific chromatin marks, followed by deep sequencing (ChIP-seq) (reviewed in^6^). ChIP-seq datasets from large-scale consortia such as those from ENCODE^7^ or the NIH Roadmap Epigenomics Mapping Consortium^8^, revealed more than 100,000 putative *c*REs that were active in embryonic and adult heart tissue in mice and humans. However, these datasets have been generated from a mixed cell population of whole organs and may not capture cREs that are only active in specific cell types.

Each cell type has a specific repertoire of *c*REs contributing to its cellular identity. To better understand and dissect the gene regulatory mechanisms involved in organ development and disease, a focus on the specific cell types constituting that organ is necessary. Cardiomyocytes are one of the critical structural and functional cell types in the heart and multiple cardiomyocyte-specific genes are known to be involved in cardiomyopathies^9,10^. Thus, elucidating the repertoire of *c*REs involved in the regulation of cardiomyocyte gene expression may be crucial for understanding the origin of cardiomyopathies and other heart related congenital diseases.

Zebrafish are an ideal model to study heart development, due to their optical transparency during development, and many shared physiological features with the human heart, such as heart rate and contractility dynamics^11^. Several studies have investigated the regulatory repertoire in adult cardiomyocytes at various stages of zebrafish development^12,13^. At 72 hours post fertilisation (hpf) the zebrafish heart has completed key developmental milestones such as tube morphogenesis, heart looping, and trabeculation, and further matures^14^. Here, we present a unique cardiomyocyte-specific repertoire of *c*REs active in the zebrafish embryonic heart at 72 hpf using a combination of transcriptomics and epigenetic profiling. We identified *c*REs associated with key genes known in several species to be essential for heart development and disease, and further validated the quality of the *c*REs repertoire by performing a transgenic assay for the *c*REs associated with *popdc2* and *bmp10* genes as cardiac regulatory elements.

## Methods

**Fig. 1:**
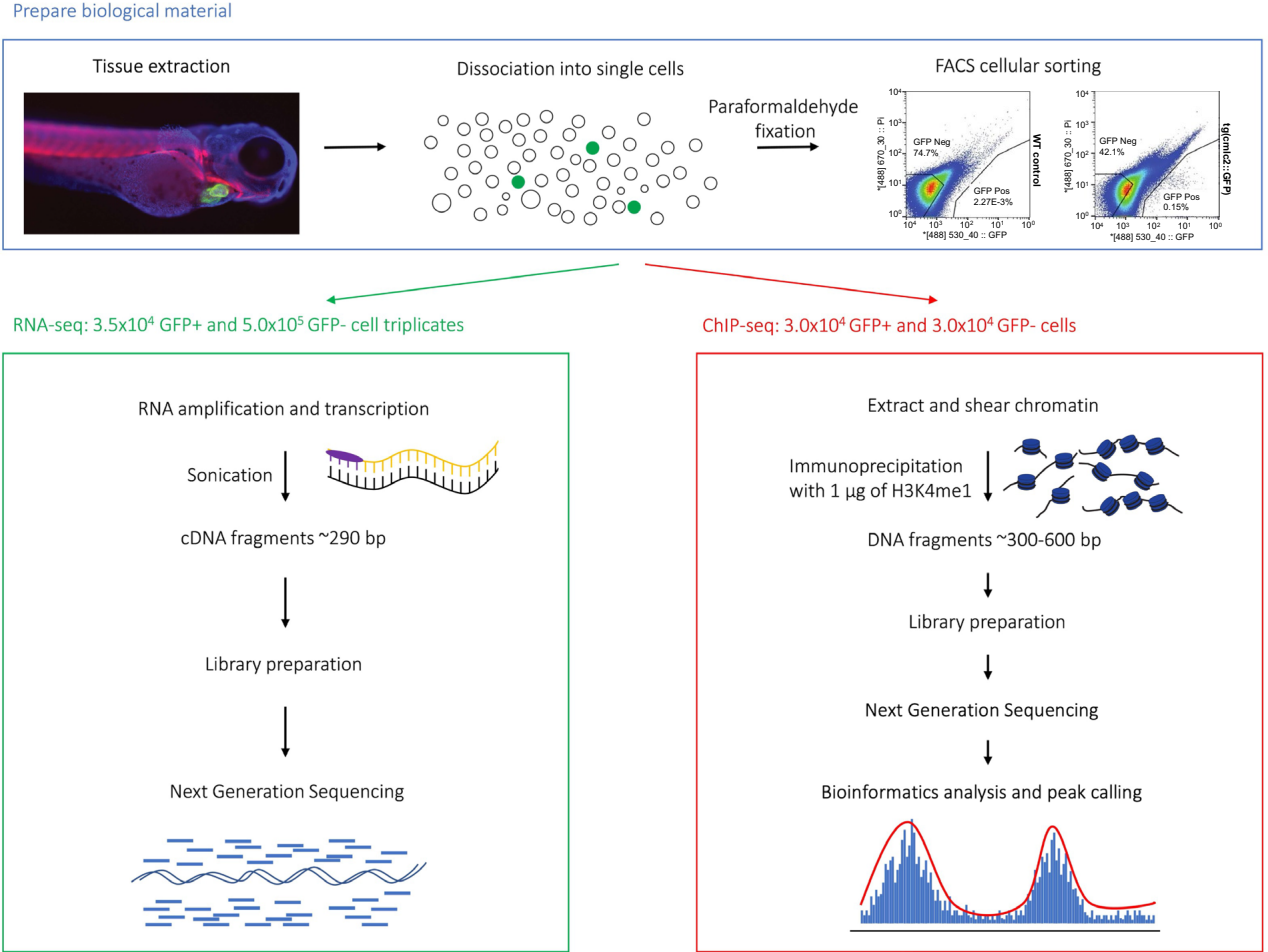
Experimental workflow for preparing and collecting zebrafish materials for next generation sequencing at 72 hpf. Tissues extracted after de-yolking were dissociated into single cells and FACS-sorted into GFP+ and GFP- pools. RNA-seq libraries were obtained from N = 6 samples, 2 conditions (GFP+ and GFP-) with 3 biological replicates. ChIP-seq libraries were obtained from N = 2 samples (GFP+ and GFP-) and N = 2 corresponding inputs. Libraries were then prepared, sequenced, and data analysed.

Biological materials from zebrafish were prepared and collected for next generation sequencing using a custom, in-house protocol (Fig. 1).

### Zebrafish husbandry

Tübingen (TU) zebrafish were maintained and bred at 26.5 °C. Embryos were raised at 28.5 °C and staged in hpf as previously described^15^. The study was carried out in accordance with the provisions of the Australian National Health and Medical Research Council code of practice for the care and use of animals.

### Dissociation

Cardiomyocytes were isolated from the cmlc2-GFP (Tg(*myl7*::GFP)) transgenic line^16^. Larvae were collected at 72 hpf, immobilised with tricaine (MS222, 200–300 mg/l, Sigma, E10521), washed with cold HBSS, and dissociated with collagenase type II (Worthington, LS004174, 100 mg/ml use 1:100 in 0.1 M TrisHCl pH 7.5) for 30 min and 0.25% trypsin (Sigma, T4049-500 ML) for 10 min at room temperature. Dissociation was facilitated by gentle, slow pipetting with a 1000 µl pipette tip. Cell suspensions were subsequently filtered through 100 µm and 40 µm nylon meshes (Falcon® Cell Strainer, Corning, 352360 and 352340) by gentle centrifugation at 2000 rpm for 5 min. Cell number and viability were assessed using an automated cell counter (Countess™, ThermoFisher Scientific, AMQAF1000). Cells were pelleted by centrifugation at 2000 rpm and resuspended in FACS buffer (1% BSA, 2% FBS in PBS).

After sorting, GFP-positive (GFP+) and GFP-negative (GFP-) cells were collected in 0.125 M glycine-PBS, frozen in liquid nitrogen and kept at −80 °C until use. Cells were pelleted by centrifugation at 2000 rpm and either snap frozen in liquid nitrogen and kept at −80 °C until use in RNA sequencing (RNA-seq) experiments or fixed with PFA (4% in PBS) and resuspended in FACS buffer (1% BSA, 2% FBS in PBS) for sorting, microscopy, and ChIP-seq experiments.

An equal volume of 4% formaldehyde (PFFA in PBS) was then added to cell suspension and cells were fixed for 10 min at room temperature. Reaction was stopped by an equal volume of ice-cold 0.25 M glycine in PBS, cells were then washed three times with 0.125 M glycine-PBS and resuspended in the same buffer. We collected a pool of 2,000–3,000 larvae to obtain the required numbers of 30,000 GFP + cells.

### Flow cytometry

72 hpf zebrafish larvae were pooled, dechorionated, dissociated into single cells, and FACS sorted. GFP + cardiomyocytes (typically 30k per sort) were compared to GFP- cells (a mix of all other cell types, typically millions of cells per sort).

Cell sorting was carried out using BD Influx Cell Sorter (BD Bioscience, 646500). Propidium iodide (10 µg/mL final concentration) was added to cell suspensions to exclude dead cells. To set the autofluorescence level, Cell Sorter was calibrated with GFP- cells before cell separation. GFP+ and GFP- cells were collected in 0.125 M glycine-PBS, frozen in liquid nitrogen and kept at −80 °C until use. For RNA-seq analysis, the PFA fixation step was omitted, and cells were sorted into complete L-15 Leibovitz medium (Gibco, 11415064) containing 20% foetal bovine serum.

All sorts were performed with a 70 µm nozzle and 60 psi sheath pressure at room temperature into low-binding tubes (Sigma, Z666505) containing 300 µl RNAlater™ Stabilization Solution (ThermoFisher Scientific, AM7020). Gates were set using an age-matched single cell suspension of wild type larvae that had been processed in parallel. To discriminate GFP + cardiomyocytes from autofluorescent body cells (e.g. pigment cells of the eye or lateral line), compensation settings were applied and only events that were GFP + but showed no fluorescence in the red channel (DsRed [561] 593/40) for propidium iodide were sorted. Cells from wildtype zebrafish (TU) were used to assign the gate threshold, so that a maximum of 0.01% of the events were considered GFP+. The fraction of GFP + events in the cell suspension of the cmlc2-GFP transgenic larvae was typically around 0.15%. Droplets that contained both a GFP+ and a GFP- event were excluded, occurring at a frequency of about 20% of GFP + events. Laser settings GFP-488 (cardiomyocytes) and PI-555 were used to exclude dead and autofluorescent cells (such as melanocytes). FACS data was analysed with the FlowJo software.

The sorted material was pelleted by centrifugation at 3000 × g. Supernatant was removed by aspiration, and the pellet was snap-frozen in liquid nitrogen and stored at −80 °C.

### RNA-sequencing

Triplicates of 3.5 × 10^4^ GFP+ and 5.0 × 10^5^ GFP- events were sorted into 300 µL RNAlater™ Stabilization Solution (ThermoFisher Scientific, AM7020). The cells were pelleted by centrifugation, snap-frozen in liquid nitrogen, and stored at −80 °C. RNA extraction was performed using the RNEasy Plus Micro Kit (Qiagen) according to the instructions provided in the manual.

Integrity of the extracted RNA was assessed with BioAnalyzer (Agilent), and the concentration was measured with Qubit (Q32866, Qubit™ RNA HS Assay Kit Q32852, ThermoFisher Scientific). Triplicates of 35,000 GFP + yielded 1–4 ng of total RNA each. Triplicates of 500,000 GFP- events contained 88–96 ng total RNA. We used the whole sample of the GFP + events and 10 ng for the GFP- events to be linearly amplified using seven cycles of single primer isothermal amplification^17^ followed by reverse transcription (Nugen Ovation RNA-seq system V2 using Nugen protocol M01206 v5, 2013). Of the resulting cDNA, 100 ng was sonicated using the Ultrasonicator to an average fragment size of ~290 bp. During the sequencing run, samples were diluted to the same concentration to prevent PCR bias. Libraries were prepared with the Ovation Ultralow System V2 following the Nugen protocol M01379 v1, 2014. Clusters were generated by c-bot clustering using 200 pM of the library pools (Illumina Protocol 15006165 v02 Jan 2016). Next-generation sequencing was performed using 50 bp single reads at Illumina HiSeq. 3000 (Illumina Protocol 15066493 Rev A, February 2015). Clustering resulted in 376.7 million reads, and 97.1% of reads had a quality score of >Q30. 58.4–66.5 million reads passed the quality filter for each sample. PhiX spike-in parameters were below the expected error rate (0.09%, expected <0.5%) and phasing/prephasing were 0.15/ <0.04 (expected <0.4/ <0.2).

### Chromatin immunoprecipitation followed by next-generation sequencing

30,000 GFP+ and 30,000 GFP- events were isolated by FACS following the dissociation and FACS-sorting protocols above with the following adaptations: after dissociation but prior to sorting, the cells were fixed with 1.8% paraformaldehyde (Sigma, #441244) in HBSS supplemented with 2% FBS at room temperature for 5 min, quenched with 0.125 M glycine final concentration (Amresco, #0167) on ice for 15 min. Live cells were sorted onto cover slips and stained with DAPI and propidium iodide to confirm that we sorted actual cells rather than just GFP + events. Although paraformaldehyde may have quenched the fluorescence, we were still able to sort a distinct fraction of GFP + cardiomyocytes. Furthermore, we supplemented PBS and the FACS buffer with 0.1% Tween-20 (Biochemicals, BIO-0777-500ML). Cells were sorted into 300 µl FACS buffer (1% BSA, 2% FBS, 0.1% Tween-20 PBS, Phosphate-buffered saline), pelleted, snap-frozen with liquid nitrogen, and stored at −80 °C. The frozen pellet of 30,000 sorted events was lysed in 100 µl lysis buffer^18^ on ice for 10 min. 30,000 sorted GFP- events were used to optimise sonication conditions. We tested 10, 20, 30, 40, 50, and 60 cycles 20 sec ON/ 30 sec OFF (Bioruptor NGS, Diagenode). The amount of DNA in the sheared samples was assessed via Qubit measurement (Invitrogen, Qubit 2.0 Fluorometer). Sheared chromatin of 30,000 sorted events contains about 30 ng DNA. Fragment size (ideally between 300–600 bp but below 1,000 bp) was determined on a BioAnalyzer (Agilent). ChIP-seq sample quality was assessed using Bioanalyzer. The Bioanalyzer traces showed that DNA fragments were at optimal lengths (Supplementary Fig. S1). Selected read length was 51, with a minimum read depth of 26,714,871 and a maximum of 34,169,233 observed in the samples.

Chromatin immunoprecipitation was performed as described in Polo *et al*.^19^. One µg of H3K4me1 antibody (ab8895, Abcam) was added to the cell lysates. Samples were incubated with magnetic beads on a wheel at 4 °C overnight. Beads had been washed for 10 min rotating on a wheel at 4 °C with the following buffers: once with dilution buffer (165 mM NaCl, 0.01% SDS, 1.1% Triton X-100, 1.2 mM EDTA pH 8.0, 16.7 mM Tris HCl pH 8.0), twice with low salt buffer (150 mM NaCl, 0.5% sodium deoxycholate, 0.1% SDS, 1% Nonidet P-40, 1 mM EDTA pH 8.0, 50 mM Tris HCl pH 8.0), twice with high salt buffer (500 mM NaCl, 0.5% sodium deoxycholate, 0.1% SDS, 1% Nonidet P-40, 1 mM EDTA pH 8.0, 50 mM Tris HCl pH 8.0), once with TE buffer (0.25 mM EDTA pH 8.0, 10 mM Tris HCl pH 8.0).

Immunoprecipitated DNA was eluted by vortexing twice for 15 min at room temperature with 100 µl elution buffer (1% SDS, 100 mM sodium bicarbonate) each. DNA was de-crosslinked by adding NaCl to a final concentration of 0.3 M with subsequent incubation at 65 °C overnight, followed by purification (Qiagen MinElute PCR Purification Kit, #28004).

Libraries were prepared using the Clontech SMARTer ChIP-seq Kit (Protocol 021115). A single equimolar pool was made based upon size-adjusted qPCR quantitation results, followed by denaturation of 14 pM used for c-bot hybridisation and cluster generation in an Illumina HiSeq. 1500 High output run (Illumina Protocol 15006165 Rev K Oct 2012). Our samples were sequenced using 50 bp single reads (Illumina HiSeq High output Mode, Illumina Protocol 15035788 Rev D, Apr 2014). Due to the low starting amount, 18 cycles of amplification were used. The ChIP-seq input was processed and sequenced in the same manner as the ChIP-seq samples.

### ChIP-seq data analysis

#### Processing of raw sequencing files

ChIP single read sequencing was performed on GFP + cardiomyocytes and GFP- cells using an Illumina Genome Analyser. FastQC was performed to check the quality of the raw reads. Poor quality reads and adapters were trimmed using Trim galore which is a wrap script developed using Cutadapt (version 1.16)^20^ and FastQC (Version 0.11.8)^21^. Illumina specific phred33 quality score cut off 28 was used with stringency of overlap with adapter sequence as 3 base pairs, and minimum read length cut off of 20 base pairs.

#### Alignment with reference genome and peak calling

Alignment of the quality filtered reads to reference genome *Danio rerio* (Genome assembly:GRCz10) Zv10 was performed using STAR (version 2.5.0a)^22^. The output SAM (Sequence Alignment/Map) file format generated, was converted to BAM and sorted and indexed. The enriched regions (or “peaks”) were identified using MACS2 (version 2.1.0.20140616)^23^ peak calling algorithm with default parameters and effective genome size as 1.50 × 10^9^ and peak type as “broad”. A comparison of peaks from the GFP+ and GFP- cells was performed to identify those specific to GFP + cardiomyocytes using in-house developed script and stored in BED file format.

#### Gene ontology

LiftOver was used to convert genome coordinates from assembly Zv10 to Zv9. Genomic Regions Enrichment of Annotations Tool (GREAT)^24^ was used to assign genes and the ontology terms associated with the peaks-associated genomic regions (assembly Zv9).

#### Data visualisation

To generate the signal density heatmap we used the deepTools 3.2.0 package, “computeMatrix”, to calculate normalised read coverage around the peak centres with 1 kb flanking regions (±1 kb). The signals were normalised to the library size, and then each H3K4me1 sample was normalised to the corresponding input. Heatmaps were generated using the function “plotHeatmap” from the deepTools 3.2.0 package.

### RNA-seq data analysis

#### Processing of raw sequencing files

FASTQ files were quality trimmed for overrepresented sequences using Cutadapt (version 1.16)^20^. The files were then processed for mapping and obtaining read counts with RNAsik^25^, using default parameters. There were two sets of counts obtained, the first were the reads mapped to genome Zv9, and the second to genome Zv10. Read counts obtained from mapping to Zv10 were used for RNA-seq analysis and visualisations. Read counts obtained from mapping to Zv9 were used for RNA-seq analysis to obtain a set of differentially expressed genes (DEGs) and were then used to overlap with the genes associated with peaks of the ChIP-seq data sets.

#### Differential gene expression analysis and functional annotation

Genes with less than 2 counts per million (cpm) in at least 3 samples were filtered out and were then normalised with the TMM scaling method^26^. Differential gene expression analysis was done with limma/voom (version 3.30.0)^27,28^ by pairwise comparison of the two different groups of cell types. A false discovery rate (FDR) <0.05 was used to filter for differentially expressed genes. David (version 6.8)^29^ was used to perform gene ontology (GO) enrichment analysis, and for the identification of transcription factors that were differentially expressed. GOPlot (version 1.0.2)^30^ was used for the visualisation of enriched GO terms, and transcription factors associated with enriched biological processes.

#### Comparisons of RNA-seq and ChIP-seq datasets

Genes associated to the peaks of the ChIP-seq datasets were overlapped with differentially expressed genes of the RNA-seq dataset. GO enrichment analysis on all the various overlaps was achieved with Metascape^31^. The overlaps between the ChIP-seq and RNA-seq datasets were visualised with Cytoscape (version 3.5.1)^32^.

#### Gene Ontology analysis

Differentially expressed genes have been associated with Gene Ontology (GO) terms^29^ and their up- or downregulation visualised.

### Transgenesis assay

#### Candidate selection

Candidate enhancer sequences were identified by intersection of the lists of genes upregulated in cardiomyocytes and the genes associated with cardiomyocyte-specific regulatory elements. *c*REs located <10 kb up- or downstream of the transcription start site and associated with the genes known to be expressed in cardiac tissue based on ZFIN database^33^ were shortlisted.

The next step was to select the enhancer sequences in UCSC Genome Browser’s GRCz11 assembly^34^. Sequences were filtered using ChIP-seq data based on whole organism analyses for H3K4me1, H3K27ac, and H3K27me3 histone modifications^35^.

GFP+ and GFP- peaks of the ChIP-seq dataset were overlapped with publicly available ChIP-seq data generated using whole zebrafish embryos H3K4me1, H3K27ac, H3K4me3, H3K36me3, and H3K27me3 histone modification peaks^35^. We prioritised conserved sequences and excluded coding as well as repetitive sequences using the UCSC Genome Browser^34^.

#### cRE cloning and *in vivo* analysis

Putative regulatory elements with ~300 bp flanking sequences were PCR amplified from genomic zebrafish DNA (Table 1) and cloned into *Sal*I and *Bam*HI sites of pTol2-mcFos-GFP reporter vector containing a minimal promoter from the mouse *cFos* gene^36,37^.

**Table 1:** Primer sequences cloned into *Sal*I and *Bam*HI to capture the putative *c*REs.

Locus	Forward primer with *Sal*I	Reverse primer with *Bam*HI	Zv9 coordinates	Zv10 coordinates
***bmp10***	*GCCGAT*GTCGACACGTTTAACAGTGAACAGTTTGT	*GCCGAT*GGATCCAAAGTGTAGTCGATTTGAACAGC	**chr5:22992242-22992934**	**chr5:20705114-20705806**
***popdc2***	*GCCGAT*GTCGACAAAACTGGAGATGAGACGAATGT	*GCCGAT*GGATCCTGAGAAACAATTGAACACATGGT	**chr9:22263074-22263569**	**chr9:21418860-21419355**

Transposase mRNA was synthesised by *in vitro* transcription using the mMESSAGE mMACHINE SP6 Kit (ThermoFisher Scientific) and purified using RNeasy Plus Micro Kit (QIAGEN). Twenty pg of the circular reporter plasmid containing the putative regulatory element and 50 pg of transposase mRNA were co-injected into one-cell stage zebrafish embryos. The developing embryos were screened at 72 hpf for GFP expression. Imaging was carried out using a Nikon C1 (Inverted/Upright) confocal microscope equipped with a 10x objective and running NIS Elements Software (Nikon, Tokyo, Japan). For the construct, about 100 embryos were injected and assayed.

A consistent GFP expression pattern observed in at least 20% of injected embryos was considered as positive. The reporter vector alone showed expression in muscles and blood cells in F0 embryos (data not shown).

## Data Records

All sequencing data (ChIP-seq and RNA-seq) have been deposited in the NCBI GEO database under the accession number GSE252152^38^. The flow cytometry data is available on Zenodo at 10.5281/zenodo.10720218^39^. The RNA-seq analyses results are available for interactive exploration at https://degust.erc.monash.edu/degust/compare.html?code=5812885a85be9051776433b1.

## Technical Validation

### Quality control of ChIP-seq libraries

**Fig. 2:**
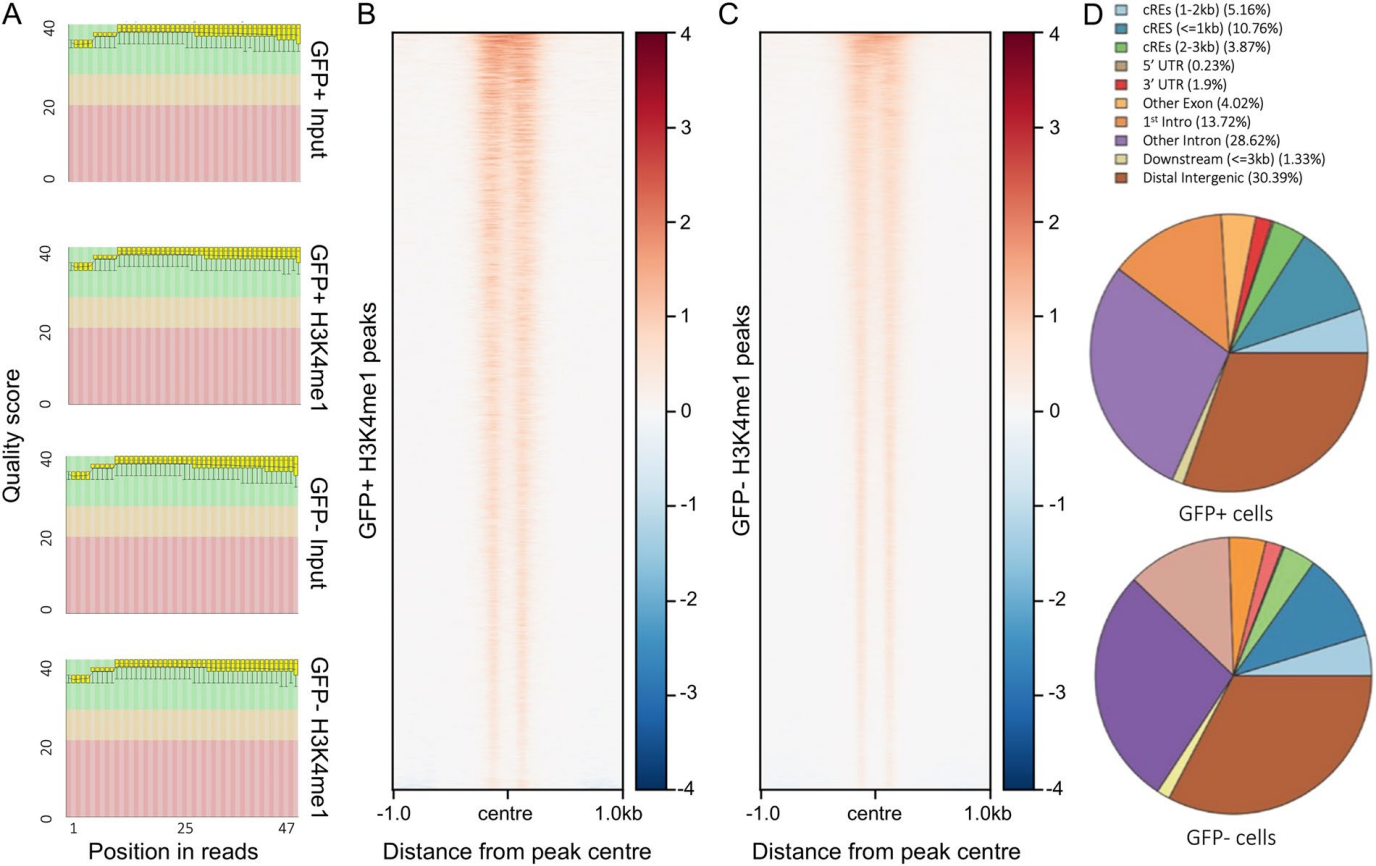
Quality metrics for ChIP-seq data. (**A**) FastQC quality control outputs for N = 2 samples (GFP+ and GFP-) and two corresponding input libraries. (**B,C**) ChIP-seq signal density heatmaps for (B) GFP + cells and (**C**) GFP- cells against the corresponding ChIP-seq inputs. All heat map densities ranged from +/−1.0 kb from the peak centre, with the average signal plots shown. (**D**) Pie charts representing the distribution of the ChIP-seq peaks in GFP+ (top) and GFP- (bottom) cells. TSS: transcription start sites; bp: base pairs; UTR: untranslated regions.

We assessed the ChIP-seq libraries using various quality scores in FASTQC (Fig. 2A, Supplementary Fig. S2). The distribution of quality scores across all bases of the reads for the ChIP-seq samples (GFP+ and GFP-) fell in the high-quality zones (Fig. 2A, green zones). We generated four H3K4me1 samples: “30k pos”, “30k pos input”, “30k neg”, and “30k neg input”. We analysed the “30k pos” sample in comparison to “30k pos input”, and similarly the “30k neg” sample in comparison to “30k neg input”. Heatmaps for the H3K4me1 peaks of the GFP+ and GFP- (Fig. 2B,C) showed a typical profile of H3K4me1 signals, with depletion at the exact centre of active enhancers where nucleosome-free regions (NFRs) are located, and heavy H3K4me1 marking immediately flanking the centre. The peak calls were made in the intergenic, 5′UTR, 3′UTR, intronic and promoter regions in both GFP+ and GFP- samples as expected (Fig. 2D).

### Quality control of bulk RNA-seq libraries

**Fig. 3:**
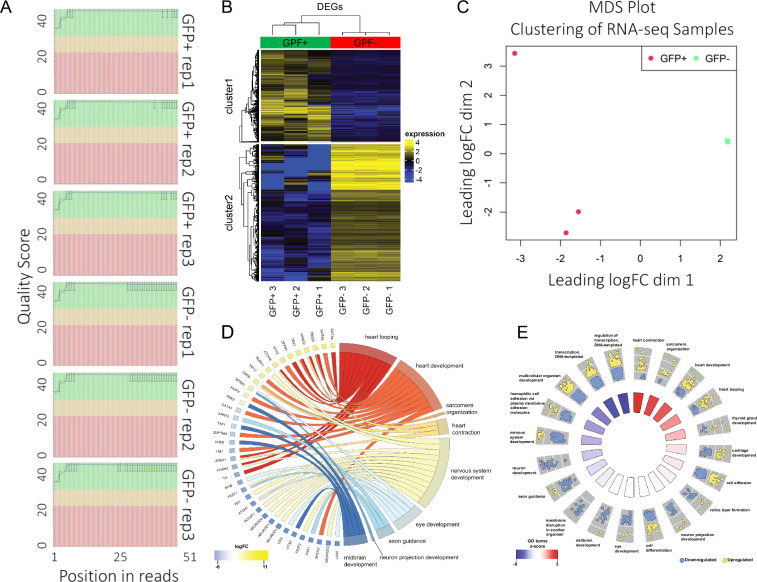
Quality metrics for bulk RNA-seq data. (**A**) FastQC quality control outputs for N = 3 samples (GFP+ and GFP−, N = 3 biological replicates). (**B,C**) Clustering analysis of the RNA-seq samples, using (**B**) heat map and (**C**) multidimensional scaling (MDS) plot visualisation (note the 3 green squares are overlapping). Heat map of all differentially expressed genes of GFP+ and GFP- cells with FDR <0.05 (no LFC cut-off). (**D**) Chord diagram (circos diagram) of differentially expressed transcription factors and their association to cardiac (heart looping, heart development, sarcomere organization, heart contraction) and neuronal/ectodermal GO terms (nervous system development, eye development, axon guidance, neuron projection development, midbrain development). Most TFs that fall into cardiac GO terms are enriched, whereas most neuronal and ectodermal TFs are depleted. (**E**) Spider plot of top 20 enriched GO terms (p-value <0.05). Upregulated and downregulated genes are indicated by yellow and blue dots in each GO term. The colour of the inner trapezoids (rectangles) indicates whether the GO term overall is enriched (red) or depleted (blue). The GO terms for heart functions are enriched, GO terms for neuronal functions are depleted.

The quality score (Q30) for all the samples showed good base quality (Fig. 3A, green zones). Clustering analyses showed two distinct clusters of cardiomyocytes-specific GFP+ and GFP- samples (Fig. 3B,C). For the RNA-seq data, the top 20 GO terms associated with the differentially regulated genes were found to be enriched in heart function including heart looping, heart development, sarcomere organization, heart contraction, and neuronal functions (Fig. 3D,E).

## Usage Notes

To study the relationship between cardiomyocyte-specific regulatory elements and predicted target genes in heart development, we have generated an interactive interface: https://ramialison-lab.github.io/pages/enhancer.html that allows biologists to query cardiomyocyte-specific regulatory elements, the associated genes, and their differential gene expression status in the RNA-seq analyses.

**Fig. 4:**
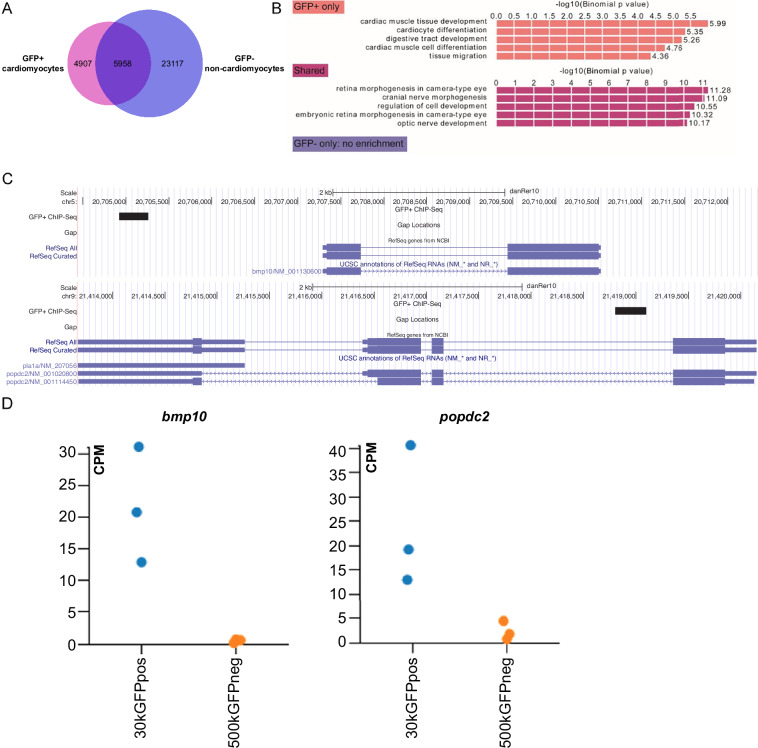
ChIP-seq and RNA-seq data for biological discovery. (**A**) Venn diagram of regulatory elements in GFP+ and GFP- cells. (**B**) GO annotations for their associated genes. GFP + only has a cardiac signature, shared *c*REs are annotated with neuronal and ectodermal functions, and GFP- show no enrichment. (**C**) UCSC ChIP-seq tracks representing the detected H3K4me1 peak (black rectangle), surrounding the *popdc2* and *bmp10* loci and (**D**) gene expression levels in the triplicate RNA-seq data (note for *bmp10*, the three orange dots are overlapping). CPM: counts per million.

We also further studied gene ontology term enrichments of both ChIP-seq data. The predicted *c*REs were assigned to genes using Genomic Regions Enrichment of Annotations Tool (GREAT) software (see Methods section). Gene ontology (GO) analysis on each population revealed that the genes associated with cardiomyocyte-specific regulatory elements were annotated with heart functions: cardiac muscle tissue development, cardiomyocyte differentiation, and cardiac muscle cell differentiation, while genes associated with regulatory elements present only in the GFP- fraction showed no heart-related enrichment (Fig. 4A,B). This data confirms that the GFP + fraction harbors regulatory elements important for cardiac function. This data can be further used to shortlist genes and enhancer candidates to study their role in heart development.

We further compared our ChIP-seq data with known heart-specific enhancers from the literature (Supplementary Fig. S4, Supplementary Table S1). Pawlak and colleagues previously performed ATAC-seq on nkx2.5+ and myl7 + cells in 72 hpf zebrafish^40^. Our ChIP-seq data had ~15% overlapping peaks the ATAC-seq peaks (Supplementary Fig. S4A). Comparing our ChIP-seq peaks with curated heart-specific enhancers on two independent studies by Yuan *et al*. and Kosicki *et al*.^41,42^, we further verified two heart-specific enhancers from our data (Supplementary Fig. S4B,C).

Another relevant application of the dataset was to test the identified cardiac regulatory elements for their putative enhancer activity, by exploring the regions with both ChIP-seq and RNA-seq signals in the zebrafish genome (Fig. 4C). Therefore, we cloned the shortlisted *c*REs associated with the *popdc2* and *bmp10* genes (see Methods) into Tol2-cFos-GFP vector and injected them into one cell stage embryos. The injected embryos were screened at 3 days post-fertilisation for GFP expression in the heart using immunofluorescence analysis. GFP expression in the heart was present in only 8.7% of control embryos, while GFP signal was detected in the hearts of 61.2% and 61.9% of the embryos injected with the vectors containing *c*REs associated with *popdc2* and *bmp10* respectively (Supplementary Fig. S5), validating these genomic regions as regulatory elements able to drive expression in the heart. The tissue-specific enhancer activity was further supported by our prior work^43^ where tissue-specific enhancer activities were demonstrated to generate *in vivo* phenotypes. To assist biologists in exploring our ChIP-seq and RNA-seq datasets, we have provided a searchable database (https://ramialison-lab.github.io/pages/enhancer.html) and interactive website for exploring the differentially expressed genes (https://degust.erc.monash.edu/degust/compare.html?code=5812885a85be9051776433b1, GSE252151, and Supplementary Table S2).

The heatmaps (Fig. 2B,C) have been normalised to library size to enable fair comparison between GFP+ and GFP- samples. While the limited sample size (n = 2 H3K4me1 samples, n = 2 inputs) precludes definitive conclusions, we hypothesise that the observed differences in signal strength (Fig. 2B,C) may reflect the enrichment of a specific cardiac cell population in the GFP + sample, resulting in more distinct and focused H3K4me1 signal patterns compared to the heterogeneous GFP- population.

Our ChIP-seq data, while having limitations including high duplicate rates and low complexity, demonstrates potential as a preliminary screening tool for identifying biologically relevant histone marks. Despite these technical constraints, our integrated RNA-seq and ChIP-seq dataset could serve as a valuable resource for other laboratories, providing initial candidate targets that warrant further validation through targeted region and/or locus-specific ChIP experiments.

## Supplementary information

Supplementary Figures S1-S5

Supplementary Table S1

Supplementary Table S2

## Data Availability

All source code used for the bioinformatics analyses and data visualisation are provided at https://github.com/Ramialison-Lab/ZebrafishHeartEnhancers/. The database is available at https://ramialison-lab.github.io/pages/enhancer.html.
