# Supplementary material for: Epigenomics and transcriptomics profiles of developing zebrafish heart cells
Source: Sci Data. 2025 Oct 7;12:1620. doi: 10.1038/s41597-025-05895-9 (PMC12504427; doi:10.1038/s41597-025-05895-9)
Supplement: Supplementary file 1 — Supplementary Figures S1-S5 [file 41597_2025_5895_MOESM1_ESM.docx]

# **Epigenomics and transcriptomics profiles of developing zebrafish heart cells**

Gulrez Chahal^1,2^^, Michael P. Eichenlaub^1^^, Markus Tondl^1^^, Michał Pawlak^3^, Monika Mohenska^1,4,5,6^, Lin Grimm^7^, Lauren Bottrell^1^, Mark Drvodelic^1^, Sara Alaei^1^, Jeannette Hallab^1^, Lisa N. Waylen^1,2^, Jose M. Polo^1,4,5,6^, Cédric Blanpain^8^, Nathan Palpant^7^, Fernando Rossello^1, 2^, Minna-Liisa Änkö^1,9^, Peter D. Currie^1^, Benjamin M. Hogan^7,11^, Cecilia Winata^10^, Ekaterina Salimova^1^, Hieu T. Nim^1,2,12*^, Mirana Ramialison^1,2,12*^

1. Australian Regenerative Medicine Institute and Systems Biology Institute Australia, Monash University, Clayton, Victoria 3800, Australia
2. Murdoch Children’s Research Institute, Royal Children’s Hospital, Parkville, Victoria 3052, Australia; The Novo Nordisk Foundation Center for Stem Cell Medicine (reNEW) Melbourne
3. Institute of Hematology and Blood Transfusion, Warsaw, Poland
4. Adelaide Centre for Epigenetics, Faculty of Health and Medical Sciences, The University of Adelaide, SA, Adelaide, 5000, Australia
5. South Australian immunoGENomics Cancer Institute, Faculty of Health and Medical Sciences, The University of Adelaide, SA, 5000, Adelaide, Australia
6. Institute for Molecular Bioscience, The University of Queensland, Brisbane, Queensland, 4072, Australia
7. Division of Genomics of Development and Disease, Institute for Molecular Bioscience, The University of Queensland, St Lucia, 4072, QLD, Australia
8. Interdisciplinary Research Institute (IRIBHM), Université Libre de Bruxelles (ULB), Bruxelles, 1070, Belgium
9. Faculty of Medicine and Health Technology, Tampere University, Tampere 33100, Finland; Hudson Institute of Medical Research, Victoria 3168, Australia
10. International Institute of Molecular and Cell Biology in Warsaw, Warsaw, 02-109, Poland
11. Peter MacCallum Cancer Centre, Melbourne, VIC 3000, Australia. Department of Anatomy and Physiology and the Sir Peter MacCallum Department of Oncology, University of Melbourne, Melbourne, VIC 3000, Australia*.*
12. Department of Paediatrics, Royal Children's Hospital, MDHS Faculty, Flemington Road, The University of Melbourne, Victoria 3010 Australia

^ equally contributing authors

* To whom correspondence should be addressed. Email: [hieu.nim@mcri.edu.au](mailto:hieu.nim@mcri.edu.au), [mirana.ramialison@mcri.edu.au](mailto:mirana.ramialison@mcri.edu.au)

# **Supplementary Figure Legends**

###
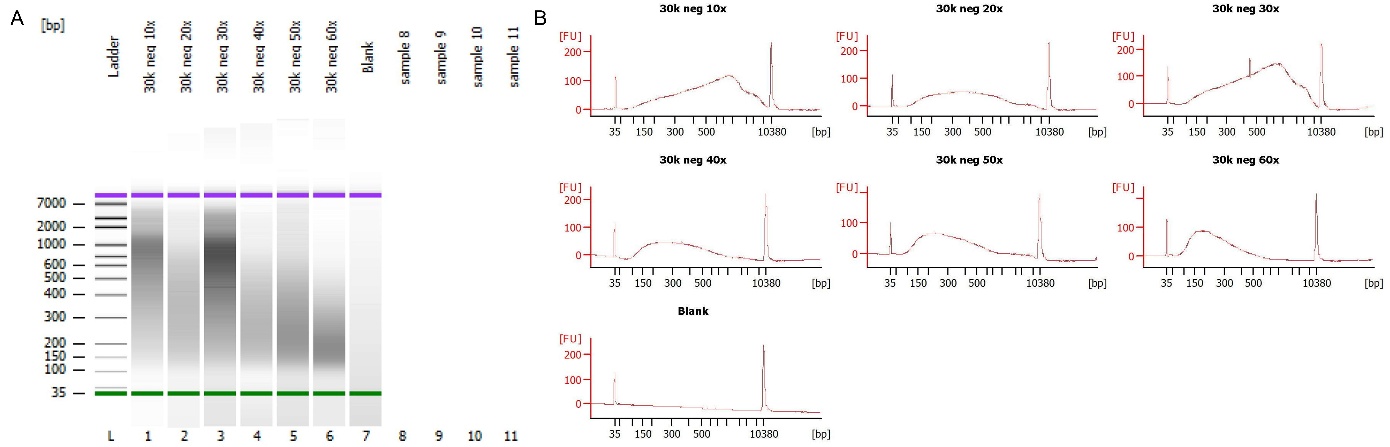


### Supplementary Figure S1. Bioanalyzer traces of the sonicated chromatin from the "30k neg" ChIP-Seq samples at 10-60 sonication cycles. (A) Gel image showing DNA fragment size distribution at 10-60 sonication cycles, and a blank control. (B) Electropherogram traces showing fluorescence intensity versus fragment size [bp] at 10-60 sonication cycles, and a blank control.


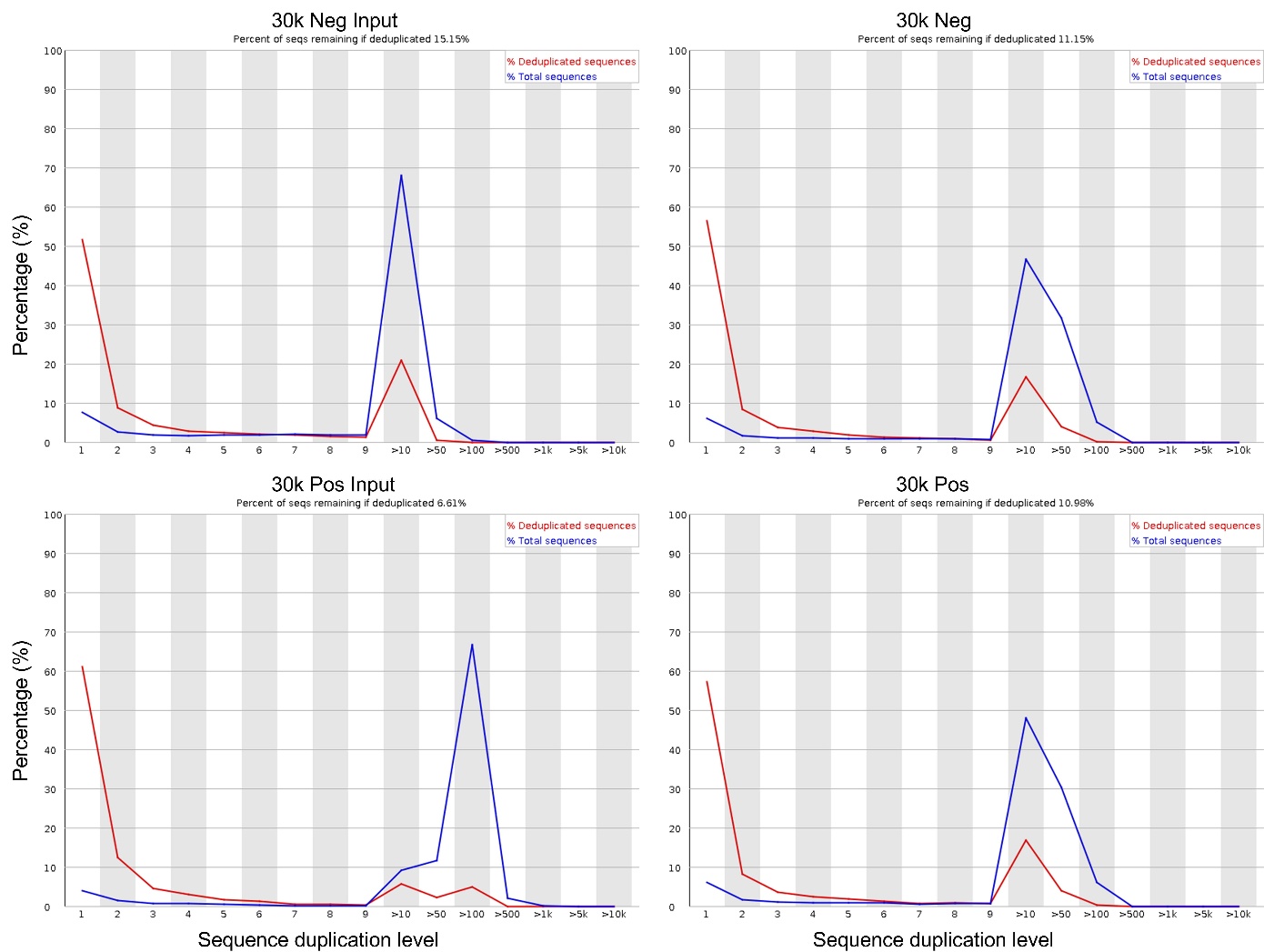


### Supplementary Figure S2. FastQC visualisation of the duplication rates of the four ChIP-Seq samples: 30k Neg Input, 30k Neg, 30k Pos Input, and 30k Pos.


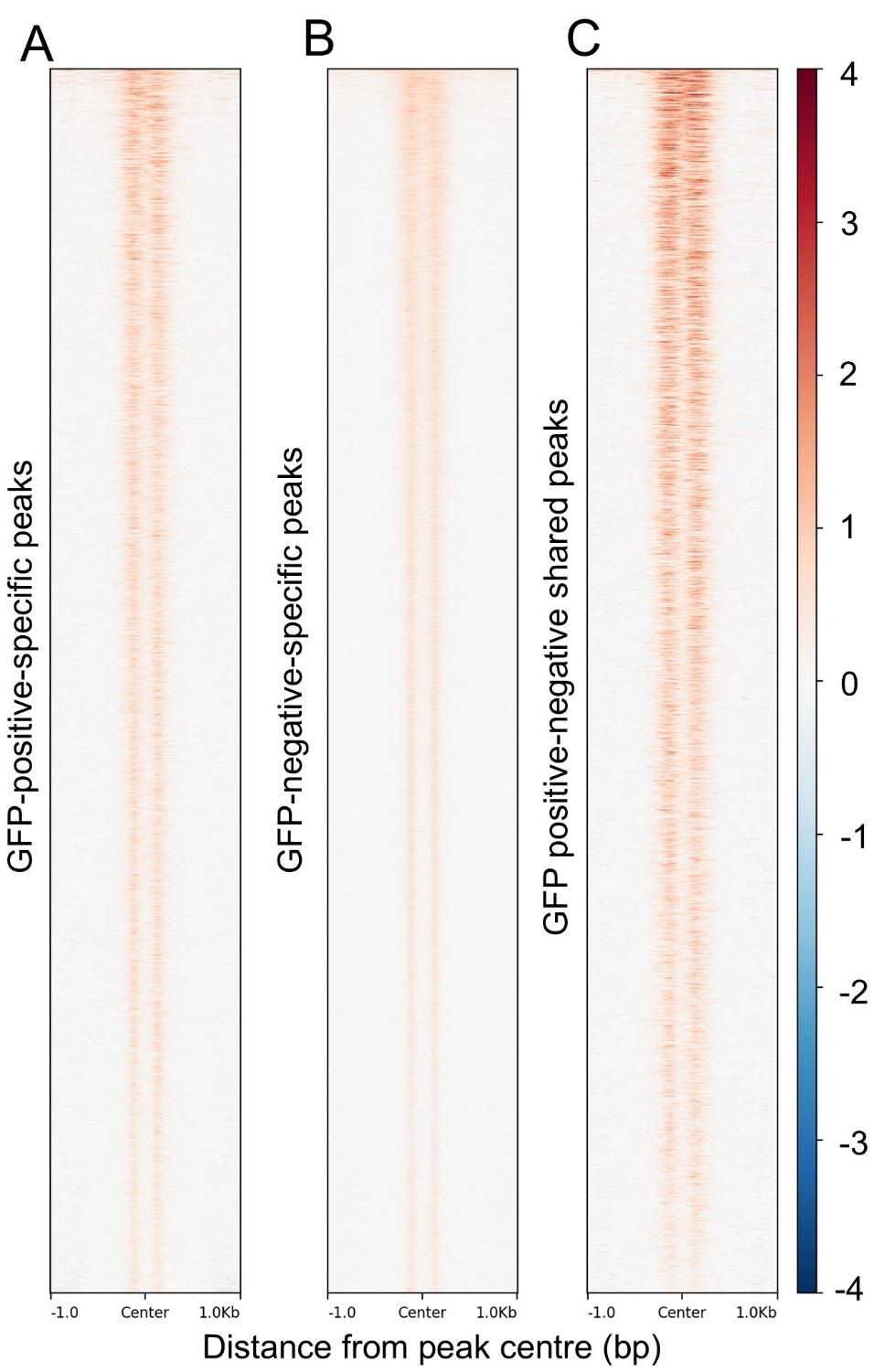


Supplementary Figure S3. H3K4me1 signal density heatmap for (A) GFP-positive-specific peaks, *i.e*. peaks present in GFP+ cells but not in GFP- cells; (B) GFP+ specific peaks, *i.e.* peaks present in GFP- cells but not in GFP- cells; and (C) GFP+/- shared peaks, *i.e*. peaks present in both GFP+ cells and GFP- cells.


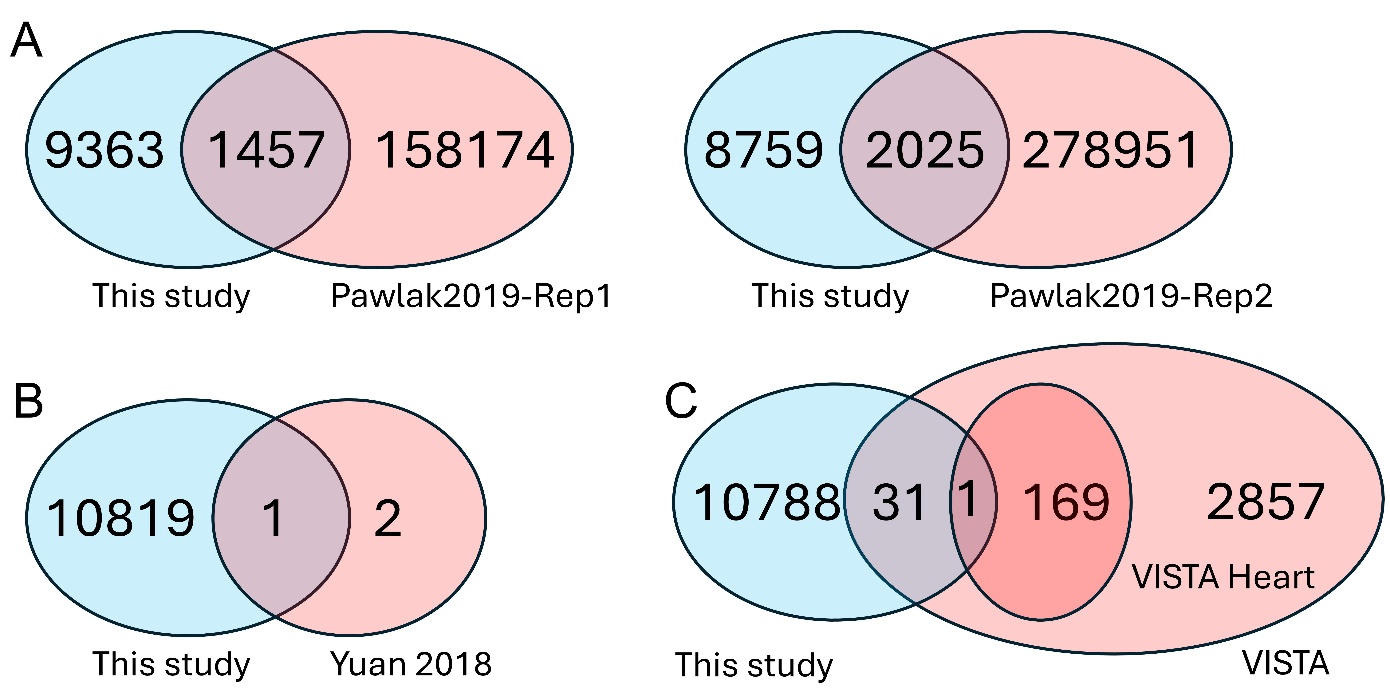


### Supplementary Figure S4. Cross comparison between the ChIP-seq peaks in this study and three other studies (A) Pawlak *et al.* 2019, (B) Yuan *et al.* 2018 and (C) Kosicki *et al.* 2024.


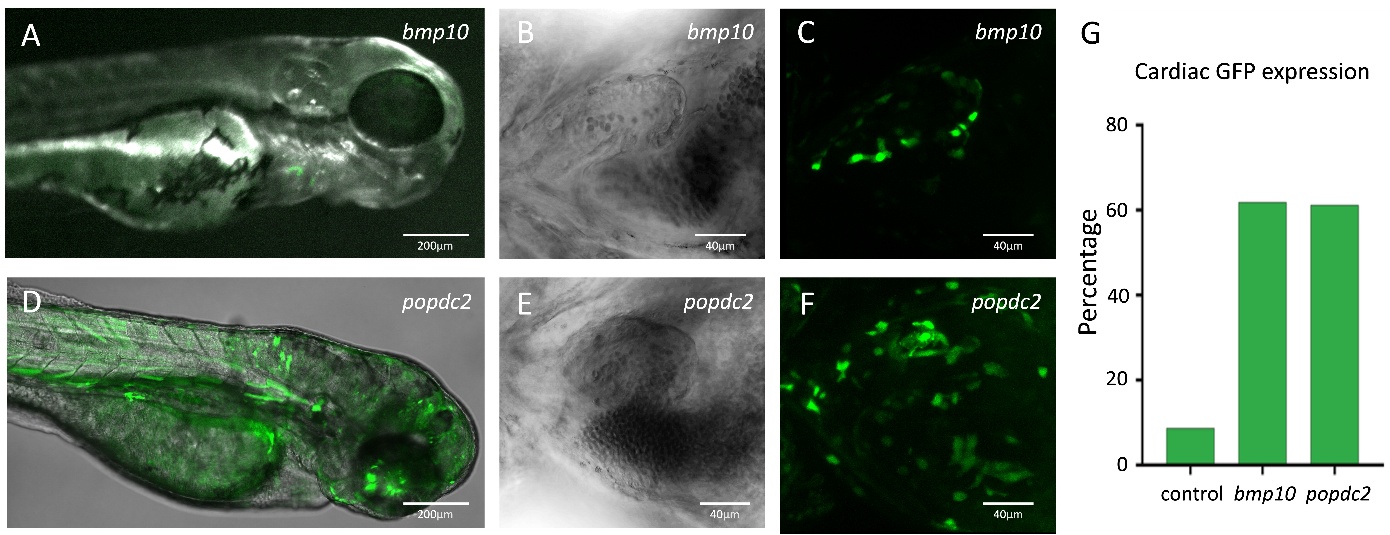


### Supplementary Figure S5. Transgenic reporter assays for the *bmp10* and *popdc2* enhancers in the larva (A-D) and heart (B-F). (G) Quantification of GFP expression detected in the heart in empty vector control lines and *popdc2* and *bmp10* regulatory element lines in F0 as a percentage of the total number of embryos injected showing any GFP expression. N=24 for control, N=63 for *bmp10* and N=49 for *popdc2*.

# **Supplementary Table Legends**

### Supplementary Table 1. Overlapping ChIP-seq peaks between this study and three other studies: Pawlak *et al.* 2019, Yuan 2018 and Kosicki 2024. Each set of overlap is represented in an Excel spreadsheet, with the “Overlap?” column marked as “TRUE” if the enhancer regions overlap with the corresponding study, and “FALSE” if otherwise.

### Supplementary Table 2. Overlaps between ChIP-seq peak-associated genes and RNA-seq differentially expressed genes in this study. The first Excel spreadsheet provides the overview, while each subsequent spreadsheet describes each overlapping subset between the ChIP-seq peak-associated gene list and RNA-seq up-regulated / down-regulated gene lists.
